# Probability matching does not decrease under cognitive load: A preregistered failure to replicate

**DOI:** 10.3758/s13421-018-0888-3

**Published:** 2019-01-07

**Authors:** Christin Schulze, Greta James, Derek J. Koehler, Ben R. Newell

**Affiliations:** 1 Center for Adaptive Rationality, Max Planck Institute for Human Development, Berlin, Germany; 2 Department of Psychology, University of Waterloo, Waterloo, ON Canada; 3 School of Psychology, University of New South Wales, Sydney, Australia

**Keywords:** Probability matching, Probability maximizing, Working memory load, *n*-back task

## Abstract

Does taxing cognitive resources improve people’s choices in repeated binary prediction? Wolford, Newman, Miller, and Wig (2004, *Canadian Journal of Experimental Psychology*, *58*, 221–228) found that a secondary verbal working memory task, which competed for cognitive resources with a repeated binary choice task, steered participants toward adopting the optimal strategy, namely, probability *maximizing*. By contrast, under single-task conditions, an inferior strategy prevailed, namely, probability *matching*. We conducted a preregistered direct replication of Experiment 1 in Wolford et al. (2004) with a sample of participants more than 5 times larger than the original sample. We did not find a statistically significant effect of cognitive load on strategy selection in repeated binary choice. Moreover, in many cases, Bayesian analyses, which were performed in addition to conventional methods of null hypothesis significance testing, yielded substantial evidence in favor of the absence of cognitive load effects on choice behavior. Thus, we found no reliable support for the claim that taxing cognitive resources leads to improved decision-making in repeated binary prediction.

Proverbial wisdom suggests that “to do two things at once is to do neither.”^1^ Yet in simple binary prediction, people’s choices have been found to *improve* when an additional task is introduced that competes for cognitive resources (Wolford, Newman, Miller, & Wig, 2004). Consider, for example, the task of predicting which of two light bulbs will illuminate over a series of trials in which one light turns on more frequently than the other (e.g., with probability .75 vs. .25). Provided these probabilities remain stationary and are serially independent, one should always pick the light bulb with the higher illumination frequency in order to *maximize probability* and prediction accuracy. Yet a common finding in the literature on repeated binary choice is that people often instead allocate their guesses in proportion to the outcome frequencies—a behavior known as *probability matching*. This strategy leads to a substantial loss in prediction accuracy (for reviews, see, e.g., Koehler & James, 2014; Newell & Schulze, 2017; Vulkan, 2000). Surprisingly, however, Wolford et al. (2004) found that, relative to participants in a control group who completed only a binary choice prediction task, participants who completed a concurrent *n*-back verbal working memory task—in which a series of numbers was concurrently presented on the screen, and participants were periodically asked to recall the last *n* numbers—showed a lower rate of probability matching (i.e., a higher rate of probability maximizing) in the binary choice task. Participants in a third group, who completed a concurrent visual-spatial memory task in which they judged randomly constructed polygons, performed much like the control group.

Wolford et al. (2004) interpreted these results as supporting the view that probability matching arises from a search for patterns in the (random) outcome sequence. They reasoned that the *n*-back task selectively taxes the cognitive resources needed for vigilant pattern search and thus undercuts this behavior (the visual-spatial task was assumed to tax cognitive functions not involved in pattern search). In other words, Wolford et al. argued that probability matching was reduced as a by-product of impeded search behavior. Yet the view that probability matching represents a cognitively sophisticated and ecologically adaptive response associated with the search for patterns (see also Gaissmaier & Schooler, 2008; Peterson & Ulehla, 1965) remains contested. In fact, there is ongoing debate among researchers about whether probability matching instead arises from cognitive constraints that motivate people to fall back on cognitively simpler heuristic choice strategies (see, e.g., Koehler & James, 2009, 2014; Kogler & Kühberger, 2007; West & Stanovich, 2003).

Wolford et al.’s (2004) results are an important and oft-cited piece of evidence in this debate, but they rest on a single experiment (Experiment 1) in which the sample size was fairly modest (*n* = 10 per condition). Moreover, other research on the role of cognitive capacity in repeated binary choice has failed to find any difference between rates of probability matching and maximizing under conditions of taxed cognitive resources versus single-task conditions (see, e.g., Otto, Taylor, & Markman, 2011; Schulze & Newell, 2016). Establishing the replicability of the original results is thus an important contribution to the debate on the causes of probability matching and the conditions under which it is more or less likely to occur.

## Preregistered replication

In this study, we sought to replicate Experiment 1 in Wolford et al. (2004). The preregistration form for the replication (Replication Recipe; see Brandt et al., 2014) is available on the Open Science Framework (https://osf.io/au3p8/).

## Method

### Participants

The planned target sample size was 150 participants, that is, *n* = 50 in each of three between-subjects conditions: single-task control, *n*-back dual task, and polygon dual task. We planned to recruit 75 participants (25 in each condition) at both the University of New South Wales, Australia, and the University of Waterloo, Canada. The planned sample size was determined via power calculation (using G*Power software) based on effect sizes estimated from the original study and the “small telescopes” approach suggested by Simonsohn (2015).^2^ Seventy-five undergraduate students from the University of New South Wales and 87 undergraduate students from the University of Waterloo participated in this experiment.^3^ Participants at each location were randomly assigned to one of the three conditions: single-task control (*n* = 57), *n*-back dual task (*n* = 54), and polygon dual task (*n* = 51). One additional participant aborted the experiment prematurely. In return for their participation, students received course credit and a small performance-based payoff; earnings ranged from AU$1.65 to AU$3.89 for Australian participants and CA$2.33 to CA$3.83 for Canadian participants. Participants’ mean age was 19.44 years (*SD* = 1.95 years, range: 17–29 years), and 110 self-identified as female.

### Materials and procedure

The instructions and experimental materials of the original study were reconstructed as closely as possible on the basis of the published journal article and correspondence with the first author. Participants were tested individually in a computer-based lab experiment, which was run on Windows computers with the screen resolution set to 800 × 600 pixels. The size of the program interface was constrained to 640 × 480 pixels. Participants in all experimental conditions completed a binary prediction task over 500 trials; for participants in the two cognitive-load conditions, the prediction task was interleaved with either a concurrent verbal (*n*-back) or visual-spatial (polygon) working memory task. Participants were instructed to be as accurate as they could on both tasks and were informed that the prediction task consisted of five blocks of 100 trials. At the end of the experiment, participants were informed about their total earnings and paid in cash.

### Binary choice prediction task

Participants were asked to predict whether a colored square would appear at the top or bottom of the screen over a series of 500 choice trials. They were informed that the sequence of outcomes was completely random. Each trial started with the presentation of either a fixation cross (in the single-task and polygon conditions) or a digit between zero and nine (in the *n*-back condition) in the center of the computer screen. The fixation cross or digit was displayed until the participant made a choice (there was no time limit) by pressing one of two designated keys on the computer keyboard (top = 9 key; bottom = comma key). After each choice, a colored square was displayed on the screen for 1 second. With a probability of *p* = .75, the square was red and appeared at the top of the screen; with 1 − *p* = .25, the square was green and appeared at the bottom. For each correct prediction, participants earned 1 cent in the local currency (AU$ or CA$, respectively), as per the incentive scheme in the original study (although there are, of course, small differences due to exchange rates between U.S., Australian, and Canadian currencies, plus inflation). After each block of 100 trials, participants were informed about their earnings and their accuracy on the prediction task (as a percentage of correct guesses) in the past block. The primary dependent measure was participants’ proportion of choices of the more probable outcome.

### Verbal working memory (*n*-back) task

In the three-back memory task that was interleaved with the prediction task, participants were asked to memorize the three numbers displayed most recently on the screen. A number between zero and nine was randomly selected and displayed in the center of the screen at the start of each choice task trial. After each choice and display of the outcome, a new digit appeared. Participants were asked to remember only the last three numbers they had seen. During each block of 100 choice trials, participants were probed four times. The probe asked participants to “please enter the last three digits:” followed by a question mark and three underlined slots (? _ _ _) in which they entered the numbers seen in the order of appearance. Participants confirmed their responses by pressing the return key. The placement of probes was random but restricted to occur between trials 11–25, 26–50, 51–75, and 76–100 of each block. Feedback on the accuracy of the answer was provided after each response. Participants were told that errors on the *n*-back memory task would reduce their earnings on the binary choice prediction task. In fact, in line with the original study, participants’ earnings were not contingent on performance in the secondary tasks.

### Visual-spatial (polygon) task

In the polygon task that was interleaved with the prediction task, participants were asked to judge whether a six-sided polygon shown in the center of the screen was the same as or different than the one shown the trial before. A blue polygon with six vertices was drawn at random for each participant before the first trial of the prediction task.^4^ On each subsequent trial, the polygon was altered with probability .50 by randomly selecting one of the six vertices and moving it 30 pixels in a randomly selected cardinal direction (N, E, S, W). The polygon was displayed until a response was made; there was no time limit for making a judgment. After each judgment, which was made by pressing a designated key on the computer keyboard (same = S key; different = D key), a 500-ms delay was inserted before presentation of the fixation cross, which served as the cue for the participant to make a prediction in the choice task. There was no trial-by-trial feedback on participants’ accuracy in the polygon task. However, after each block of 100 trials of the choice task, participants were informed about the proportion of correct judgments on the polygon task in that block (in percentages). Participants were told that errors on the polygon task would reduce their earnings on the binary choice prediction task. In fact, in line with the original study, participants’ earnings were not contingent on performance in the secondary tasks.

### Preregistration and data analyses

The preregistration form (Replication Recipe; Brandt et al., 2014), the instructions given to participants, and the actual program used to run the experiment were preregistered on the Open Science Framework on February 16, 2015, and can be found at https://osf.io/au3p8/. Data collection started on August 12, 2015, and concluded on September 21, 2016; all data are available at https://osf.io/hemp4/. As far as possible, we followed the analysis plan reported in the original publication. Additionally, we conducted Bayesian inference, based on Bayesian ANOVAs (Rouder, Morey, Speckman, & Province, 2012), Bayesian *t* tests (Rouder, Speckman, Sun, Morey, & Iverson, 2009), and Bayesian contingency analyses using independent multinomial sampling (Jamil et al., 2017). For these analyses, we report Bayes factors that quantify how much more likely it is for the data to have occurred under one hypothesis than another. All Bayes factors were estimated in JASP (Version 0.8.4; JASP Team, 2018), which, for Bayesian ANOVAs, provides inclusion Bayes factors (denoted as *BF*_Inclusion_) that quantify the strength of evidence for the presence of a particular effect averaged across models that include that effect (Rouder, Morey, Verhagen, Swagman, & Wagenmakers, 2017; Wagenmakers et al., 2017). For all remaining Bayesian analyses, we report Bayes factors that quantify the strength of evidence in favor of the alternative hypothesis (denoted as *BF*_10_), where *BF*_10_ > 1 indicates support for the alternative hypothesis and *BF*_10_ < 1 indicates support for the null hypothesis. Conventionally, a *BF*_10_ between 3 and 20 (0.33–0.05) is interpreted as indicating positive evidence for the alternative (null) hypothesis, 20 to 150 (.05–.0067) as strong evidence, and greater than 150 (<.0067) as very strong evidence (Kass & Raftery, 1995). For brevity, extremely large Bayes factors are reported as *BF* > 100,000.

A successful replication was defined as (a) a greater probability maximization rate (lower matching rate) on the final block of 100 trials in the *n*-back dual-task condition than in the single-task control condition (critical test); (b) an interaction between condition (*n*-back dual-task vs. single-task control) and trial block; and (c) a lack of relationships (a) and (b) for the comparison between the polygon dual-task and single-task control condition. We used three approaches to evaluate the success of the replication on the critical test: First, we determined whether our results revealed a statistically significant effect in the same direction as the original study (*n*-back dual-task > single-task control) and whether the original effect size (estimated at *d* = 1.09) was within the 95% CI of the effect-size estimate we obtained (see, e.g., Open Science Collaboration, 2015). Second, we compared the 95% CI of the replication effect size with an effect size that would give only 33% power to the original study to determine whether the replication obtained an effect large enough to have been detectable with the original sample size (based on the original sample size in Wolford et al., 2004, this effect size *d*_33%_ = .72; see Simonsohn, 2015). Third, we took a Bayes factor approach to quantify the degree to which our data support either the null or the original hypothesis. This multipronged approach is in line with the current debate on best practice in replication studies, which suggests that robust inferences across multiple approaches and the combined use of both frequentist estimation and Bayesian inference are more likely to yield defensible conclusions (Maxwell, Lau, & Howard, 2015; Simonsohn, 2015; Zwaan, Etz, Lucas, & Donnellan, 2017).

In addition to the preregistered analyses mirroring those of the original study, we conducted further robustness checks as well as exploratory analyses regarding the experiment location (Australia or Canada), for which we had no prior hypotheses. These auxiliary analyses are reported in the *Exploratory analyses* section below.

## Results

### Prediction task performance

**Fig. 1:**
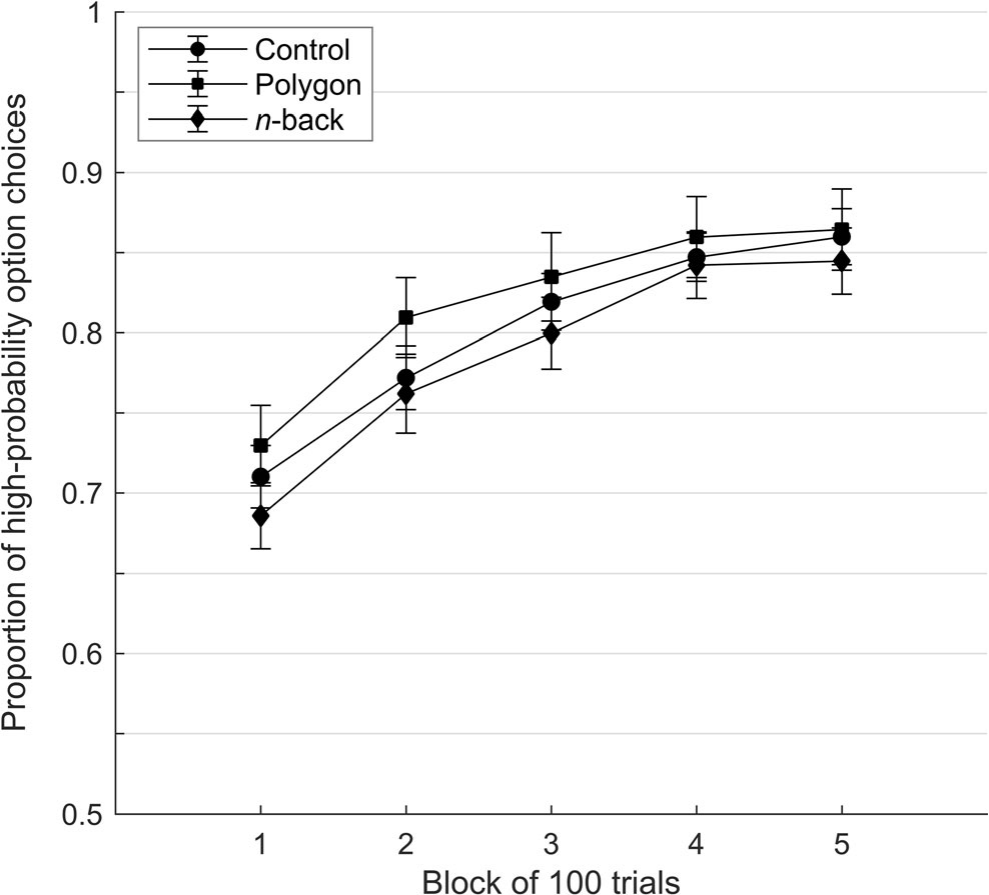
Mean (± standard error) proportion of participants’ choices of the high-probability option in each block of 100 choice trials and each experimental condition

The proportions of participants’ choices of the high-probability option across each block of 100 trials and for each cognitive load condition (see Fig. 1) were subjected to a 3 (condition) × 5 (block) mixed-model ANOVA. The main effect of learning across trial blocks was significant, *F*(3.105, 493.617) = 87.886, *p* < .001, ƞ_p_^2^ = .356, *BF*_Inclusion_ > 100,000, and is illustrated by the upward trajectory of all group lines in Fig. 1. Neither the main effect of condition, *F*(2, 159) = 0.692, *p* = .502, ƞ_p_^2^ = .009, *BF*_Inclusion_ = 0.153, nor the condition by block interaction, *F*(6.209, 493.617) = 0.518, *p* = .801, ƞ_p_^2^ = .006, *BF*_Inclusion_ = 0.002, was significant; in fact, the Bayesian analysis provided evidence against both effects. The critical follow-up *t* test indicated that the proportion of choices of the high-probability option in the final trial block did not differ between the *n*-back dual-task and single-task control groups, *t*(109) = −0.562, *p* = .576, *d* = −0.107, 95% CI for *d =* [−0.479, 0.266], *BF*_10_ = 0.232. In fact, all evaluation criteria we applied for the critical test point to a failure to replicate the original result. Specifically, using frequentist estimation, we found that the focal effect was not statistically significant (and not in the same direction as in the original study) and that the 95% CI of the effect size estimate we obtained in the replication excluded both the original effect size (estimated at *d* = 1.09) and the small effect size that would give only 33% power to the original study (*d*_33%_ = .72; see Simonsohn, 2015), suggesting that the original study could not have meaningfully examined an effect that small.^5^ Using Bayesian inference, we found that the data are 4.31 times more likely to have occurred under the null than the alternative hypothesis.

Comparing the proportion of choices of the high-probability option in the final trial block between the *n*-back dual-task and polygon dual-task groups, we again found no significant difference, *t*(103) = −0.604, *p* = .547, *d* = −0.118, 95% CI for *d =* [−0.501, 0.265], *BF*_10_ = 0.243. Finally, for the penultimate trial block, the effect sizes were smaller for both the *n*-back dual-task versus single-task control comparison (*d* = −0.037; 95% CI [−0.409, 0.335]) and the *n*-back dual-task versus polygon dual-task comparison (*d* = −0.105; 95% CI [−0.488, 0.278]).

**Fig. 2:**
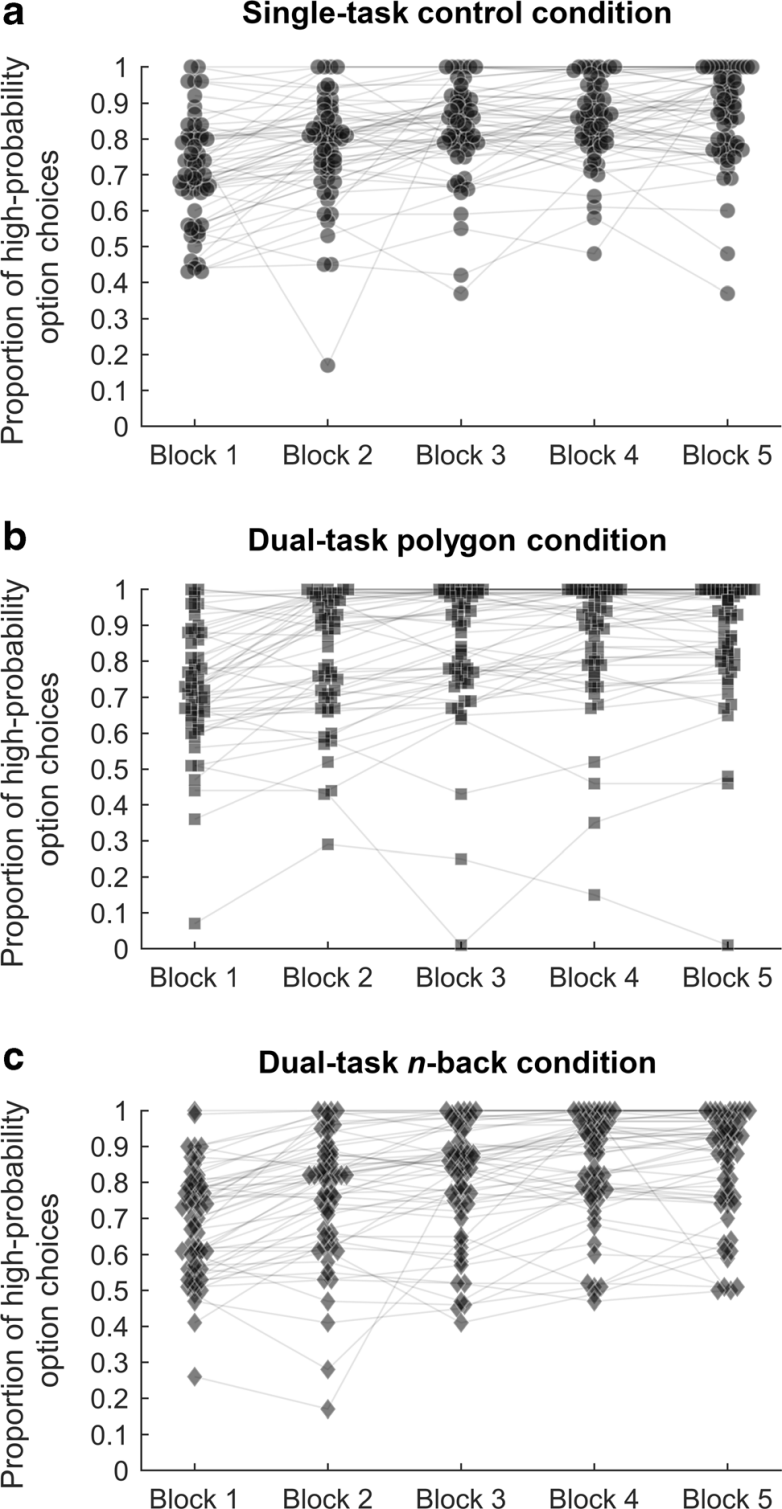
Distribution of participants’ proportions of choices of the high-probability option across each block of 100 trials for (**a**) the single-task control condition, (**b**) the dual-task polygon condition, and (**c**) the dual-task *n*-back condition. Each shape represents the choice proportion of one participant during a particular trial block and in the specified condition. Gray lines connect the choice proportions of individual participants across blocks

Figure 2 displays the full range of individual participants’ choice proportions for all trial blocks and conditions. To assess strategy selection in individual participants toward the end of learning, Wolford et al. (2004) defined maximizing as choosing the high-probability option on no less than 95% of trials in each of the last two blocks. In our study, the choice proportions of 18 participants in the polygon dual-task condition, 15 participants in the *n*-back dual-task condition, and 10 participants in the single-task control condition met this definition of maximizing; the association between probability maximizing and choice condition was not significant, *χ*^2^(2) = 4.413, *p* = .110, *BF*_10_ = 0.344. Defining maximizing as choosing the high-probability option on no less than 95% of trials in the final block (e.g., Newell & Rakow, 2007), we found that 22 participants in the polygon dual-task condition, 17 participants in the *n*-back dual-task condition, and 18 participants in the single-task control condition were classified as maximizers, *χ*^2^(2) = 2.064, *p* = .356, *BF*_10_ = 0.121.

### Secondary task performance

Table 1 shows participants’ performance on the secondary tasks for each block and each of the two dual-task conditions. Specifically, the table summarizes participants’ mean proportion of correctly recalled digits in each block of the verbal working-memory (*n*-back) task and the mean proportion of correct judgments about whether the polygon shown was the same as or different than the one shown in the previous trial for each block of the visual-spatial (polygon) task. Across all trials, participants were significantly more accurate on the *n*-back task (*M* = .849) than on the polygon task (*M* = .651), *t*(103) = 7.823, *p* < .001, *d* = 1.528, 95% CI for *d =* [1.089, 1.960], *BF*_10_ > 100,000. There was no correlation between accuracy on the secondary tasks and the proportion of choices of the high-probability option averaged across the last two blocks, *r*(103) = .054, *p* = .582, *BF*_10_ = 0.142.

**Table 1:** Mean (*SD*) response accuracy on the secondary tasks in each block of 100 choice trials

	*n*-back	Polygon
Block 1	.769 (.221)	.675 (.100)
Block 2	.898 (.166)	.665 (.077)
Block 3	.880 (.206)	.641 (.079)
Block 4	.863 (.204)	.634 (.084)
Block 5	.838 (.226)	.639 (.102)

### Exploratory analyses

The analyses reported above directly reproduce the experimental protocol of the original study conducted by Wolford et al. (2004) and utilize data from all participants we tested. These analyses failed to replicate an effect of cognitive load on strategy selection in repeated binary choice. We went to considerable lengths to conduct a well-powered, close replication of the original study; nevertheless, slight methodological differences between the original study and our replication may have contributed to our failure to replicate the original result. In this section, we therefore report supplementary analyses aimed at establishing the robustness of our conclusions. These analyses are purely exploratory, and we had no theory-based, a priori reason to anticipate systematic effects.

Figure 2 shows that some participants strictly maximized probability from the very first block of 100 trials and continued to do so throughout the entire experiment; that is, they never once selected the low-probability event. As we would have expected at least some period of probability learning before the adoption of a maximizing strategy, this finding might indicate that some participants had prior knowledge about the purpose and design of the task. Moreover, our final sample size slightly overshot the planned sample size, resulting in a somewhat unbalanced design. Addressing these issues, we reran all data analyses, restricting the sample to the planned 150 participants (*n* = 50 per condition, 75 per location), excluding participants who maximized in the first block (at least 95% choices of the high-probability option; *N* = 148), or applying both of these criteria (*N* = 136). None of these restrictions changed the conclusions reported above. That is, the ordinal patterns of choices across conditions remained largely the same (or collapsed virtually on top of each other), significant/insignificant *p* values remained either significant or insignificant, and, where *BF*s indicated positive evidence in favor of/against the presence of an effect, this continued to be the case (except for the chi-squared test assessing the association between probability maximizing, as defined in Wolford et al., 2004, and choice condition, for which previously inconclusive Bayesian evidence in favor of the null hypothesis became more substantial when we restricted the analysis to the 150 participants originally planned).

Finally, we included experiment location (Australia or Canada) as an additional factor in the mixed-model ANOVA on participants’ choices of the high-probability option, allowing us to explore potential differences between nationalities. This analysis revealed a significant main effect of location, *F*(1, 156) = 6.209, *p* = .014, ƞ_p_^2^ = .038, *BF*_Inclusion_ = 3.994, and a location by condition interaction, *F*(2, 156) = 6.178, *p* = .003, ƞ_p_^2^ = .073, *BF*_Inclusion_ = 4.716. Neither the main effect of condition nor any of the other interactions including the condition factor reached statistical significance (all *p*s ≥ .296; all *BF*_Inclusion_ ≤ 1.079). Moreover, analyzing the data obtained from each location separately,^6^ we found that neither of the two data sets provided evidence for the effect we attempted to replicate. When we focused on Australian participants only, neither the main effect of condition, *F*(2, 72) = 1.148, *p* = .323, ƞ_p_^2^ = .031, *BF*_Inclusion_ = 0.326, nor the condition by block interaction, *F*(5.796, 208.670) = 0.707, *p* = .639, ƞ_p_^2^ = .019, *BF*_Inclusion_ = 0.027, was significant, and choice proportions in the final trial block did not differ between the *n*-back dual-task and single-task control group, *t*(48) = 1.748, *p* = .087, *d* = 0.494, 95% CI for *d =* [−0.071, 1.055], *BF*_10_ = 0.973, although the ordinal pattern of choice proportions approximated that of the original study (*n*-back dual-task > single-task control ≈ polygon dual-task). By contrast, for the Canadian sample, the main effect of condition in the mixed-model ANOVA on participants’ choices of the high-probability option was significant, *F*(2, 84) = 7.941, *p* < .001, ƞ_p_^2^ = .159, *BF*_Inclusion_ = 30.854, but participants in the *n*-back dual-task condition maximized significantly *less* during the final trial block than participants in the single-task control condition, *t*(59) = −2.615, *p* = .011, *d* = −0.670, 95% CI for *d =* [−1.185, −0.151], *BF*_10_ = 4.266, or participants in the polygon dual-task condition, *t*(53) = −2.732, *p* = .009, *d* = −0.738, 95% CI for *d =* [−1.282, −0.187], *BF*_10_ = 5.408. The condition by block interaction was not significant, *F*(6.130, 257.463) = 1.078, *p* = .376, ƞ_p_^2^ = .025, *BF*_Inclusion_ = 0.165.

## Discussion

We were unable to replicate the beneficial effect of cognitive load on maximization in repeated binary choice reported by Wolford et al. (2004, Experiment 1). We had predefined a successful replication as (a) a greater probability maximization rate on the final block of 100 trials in the *n*-back dual-task condition than in the single-task control condition; (b) an interaction between condition (*n*-back dual-task vs. single-task control) and trial block; and (c) a lack of relationships (a) and (b) for the comparison between the polygon dual-task and single-task control condition. Based on data from 162 participants—a sample size more than 5 times that of the original study—our preregistered replication study revealed no statistically significant effects of cognitive load on strategy selection in repeated binary choice according to any of the measures we preregistered. Moreover, multiple evaluation criteria, including both frequentist and Bayesian approaches, used to make the critical comparison between choices in the *n*-back dual-task and single-task control conditions toward the end of learning consistently supported the conclusion that our replication was unsuccessful.

We went to considerable lengths to reconstruct the original study as closely as possible based on the published journal article and correspondence with the first author. Nevertheless, unavoidable minor differences in materials, populations, and procedures between our study and the original may have had an unpredictable impact on the replicability of the results—although we could think of no theory-driven reasons to anticipate systematic effects on the results a priori. The one possible exception is the secondary polygon task, for which we had the least information to guide our reconstruction attempt. Indeed, the results indicate that our reconstruction of this task may have been substantially more difficult to perform concurrently than the original (65% vs. 75% judgment accuracy in the replication and original study, respectively). Importantly, there were no differences in accuracy between the original and replication study on the *n*-back memory task (85% in both studies). Moreover, the stimuli were very simple (colored squares, digits, shapes) and—although the original study was conducted in the United States but the replication in Australia and Canada—participants in both studies were undergraduate psychology students and both experiments were conducted in English. Thus, we believe that these differences played only a minor role, if any, in the replicability of the effect of working memory load on binary choice reported in the original study.

Our findings suggest that an effect of cognitive load on maximizing consistent with our data would have been undetectably different from zero with the original sample size (*n* = 10 per condition; see Simonsohn, 2015). This conclusion is supported by other studies that failed to find any differences in choice behavior under conditions of taxed cognitive resources versus single-task conditions (see, e.g., Otto et al., 2011; Schulze & Newell, 2016). This prior research has suggested that rather than facilitating optimal maximizing, cognitive load may instead impact the cognitive processes underpinning people’s decisions. In particular, it has been suggested that taxed cognitive resources may limit people’s ability to track their own choices—rather than their ability to track patterns in the outcome sequence—and thus lead to the use of more easily implementable strategies (Schulze & Newell, 2016). Using computational modeling, Otto et al. (2011) found that cognitive load reduced people’s sensitivity to current outcomes and led to reliance on longer windows of past experience. These findings are in line with recent criticism of the notion that human cognition could benefit from explicit cognitive effort being withdrawn in favor of implicit processing (e.g., Newell, 2015) and with recent computational modeling approaches identifying choice errors—rather than qualitative shifts in preferences—as the primary consequence of a reduction in cognitive resources (Olschewski, Rieskamp, & Scheibehenne, 2018). In sum, our results do not support the idea put forward by Wolford et al. (2004) that working memory load improves performance in simple repeated choice by undercutting people’s tendency to search for patterns in (random) outcome sequences.

## Footnotes

1 Attributed to the Roman slave Publius Syrus (see Lyman, 1856).

2 Specifically, based on the information displayed in Figure 1 in Wolford et al. (2004)—which shows the proportions of participants’ choices of the more likely outcome in each block of 100 trials and each of the three cognitive-load conditions—we estimated means and standard errors for each block and each condition. Because the difference between conditions emerged only after participants had had the opportunity to learn the relevant outcome probabilities, Wolford et al. focused on the interaction between choice block and condition, and conducted independent-samples *t* test comparisons between conditions in the final two blocks of 100 choice trials. Because the interaction statistics are difficult to reproduce from their Figure 1 (and the effect size itself is not reported in the article), we simplified our power calculations by focusing on independent-samples *t* test comparisons between conditions for the final block of 100 trials. The effect size for the critical comparison between the *n*-back dual-task and single-task control conditions was estimated as *d* = 1.09; the effect size for the comparison between polygon dual-task and single-task control conditions was estimated as *d* = −0.48 (the sign is negative because the maximizing rate was lower in the polygon than in the control condition). For an independent-samples *t* test (one-tailed with alpha = .05) comparing *n*-back and control conditions, the power calculation gives a power of .98 for *n* = 25 per condition, suggesting that even if the Australian and Canadian samples were analyzed separately, the replication study would be sufficiently powered. The effect size estimated from Wolford et al.’s results may of course be an overestimate (e.g., due to publication bias; see Lane & Dunlap, 1978); combining data from the two samples allows us to attain sufficient power to detect even a substantially smaller effect size (e.g., for an effect size of only *d* = 0.50, the same *t* test would have a power of .80 with *n* = 50). Moreover, the target sample size of 50 subjects per condition (as well as the smaller sample size at each location) also satisfies the criterion of Simonsohn’s (2015) “small telescopes” approach, which requires a sample size at least 2.5 times that of the original study to achieve sufficient statistical power (approximately .80) to reject the null hypothesis of a detectable effect for the original sample size if the true effect is zero.

3 The slightly higher number of participants at the University of Waterloo was due to an unexpectedly high turnout rate during recruitment.

4 The size of each polygon was constrained so that, initially, all vertices were at least 210 pixels away from all screen boundaries (and no lines crossed). Similarly, all subsequent randomly occurring changes to the polygon were constrained to not cause sides to intersect or vertices to touch the edges of the screen.

5 In fact, the upper bound of the confidence interval we obtained suggests that the original study was, at best, powered to a very low 8.7%.

6 As outlined in footnote 2, analyzing the samples from the two locations separately still satisfies the criterion of a sample size at least 2.5 times that of the original study (Simonsohn, 2015) and, based on estimation of the original effect size, suggests a power of .98 for the critical comparison between *n*-back dual-task and single-task control condition in the final trial block via an independent-samples *t* test.
